# Association Between Antidepressant Use and Hemoglobin A1c Levels in Adults With Comorbid Type 2 Diabetes Mellitus and Depression

**DOI:** 10.7759/cureus.87442

**Published:** 2025-07-07

**Authors:** Oluwabukola C Oyeleye-Adegbite, Kingsley O Ozojide, Omotola Akinade, Feyisayo O Oguntuase, Izuchukwu E Okeji, Joy A Odedele, Oluwagbeminiyi M Adepeko, Stella Ehi Egege, Okelue E Okobi

**Affiliations:** 1 Public Health, Texas A&M University, College Station, USA; 2 Public Health, Nottingham Trent University, Nottingham, GBR; 3 Internal Medicine, General Hospital Ikorodu, Lagos, NGA; 4 General Medicine, National Pirogov Memorial Medical University, Vinnitsa, UKR; 5 General Medicine, North Cumbria Integrated Care, NHS Foundation Trust, North Cumbria, GBR; 6 Family Medicine, Obafemi Awolowo University Teaching Hospital Complex, Osun, NGA; 7 General Medicine, Obafemi Awolowo University, Ile-Ife, NGA; 8 Family Medicine, International University of the Health Sciences (IUHS), Basseterre, KNA; 9 Family Medicine, IMG Research Academy &amp; Consulting LLC, Homestead, USA; 10 Family Medicine, Larkin Community Hospital Palm Springs Campus, Miami, USA; 11 Family Medicine, Lakeside Medical Center, Belle Glade, USA

**Keywords:** antidepressants, depression, glycemic control, hba1c, nhanes, survey-weighted regression, type 2 diabetes

## Abstract

Background: Type 2 diabetes mellitus (T2DM) and depression commonly co-occur, and antidepressants are frequently prescribed. However, their potential impact on glycemic control remains unclear.

Objective: This study aimed to evaluate the association between antidepressant use and hemoglobin A1c (HbA1c) levels among U.S. adults with comorbid T2DM and depression.

Methods: This study used data from the 2005-2018 National Health and Nutrition Examination Survey (NHANES). Adults with both T2DM and depression were included. Descriptive statistics and survey-weighted multivariable linear regression were used to assess the relationship between antidepressant use and HbA1c, adjusting for age, gender, race/ethnicity, and BMI.

Results: Among 11,141,019 weighted individuals, 6.7% reported antidepressant use. No significant difference in HbA1c levels was found between users and non-users of antidepressants (adjusted beta (β) = 0.091, p = 0.118). Age was inversely associated with HbA1c, while BMI showed a modest positive relationship.

Conclusion: Antidepressant use was not significantly associated with HbA1c levels in U.S. adults with T2DM and depression. Further research is needed to explore the effects of specific antidepressant types and behavioral factors on glycemic control.

## Introduction

Type 2 diabetes mellitus (T2DM) and depression are two of the most widespread chronic conditions worldwide, and when they happen together, they cause significant problems for public health. About one out of every four people with T2DM in the United States is also thought to experience depression at some point [1]. The common issues experienced by people with these diseases result in more difficulties managing their health, poorer glycemic outcomes, increased risk of complications, less use of medication, and more visits to the doctor [2, 3]. Therefore, it has been discovered that having diabetes or depression can affect and worsen each other [4]. Depression makes it harder for someone to look after their diabetes, and diabetes can increase a person’s depressive symptoms [5].

Antidepressant treatment is still at the heart of the clinical management of depression. Most often, doctors treat patients with depression and diabetes using selective serotonin reuptake inhibitors (SSRIs), serotonin and norepinephrine reuptake inhibitors (SNRIs), or other antidepressant medicines [6]. Nevertheless, there is some worry about possible effects on metabolism due to some antidepressants [7]. Certain medications are known to alter appetite, lead to weight gain, lower sensitivity to insulin, and impact glucose metabolism, each of which can seriously affect blood sugar levels [8, 9]. As hemoglobin A1c (HbA1c) is the usual marker for checking long-term blood sugar in diabetics, it is necessary to learn how antidepressants may change HbA1c [10].

Research on the link between using antidepressants and controlling diabetes has produced results that are not always the same. Certain antidepressants, such as the SSRI fluoxetine, might help improve glucose control for people with diabetes by boosting mood and encouraging them to manage their condition according to routine [11, 12]. In contrast, some have found that tricyclic or atypical antidepressants can increase HbA1c levels, possibly because they boost appetite or make people gain weight [13]. These results suggest that more population studies must take into account what kind of medication was given, for what length of time, and what kind of patients received it [14].

Additionally, demographic factors such as individuals’ age, gender, economic status, and medical background could also influence both the decision to give antidepressants and the result of diabetes. There are still differences in care for mental health and diabetes based on people’s race and ethnicity [15, 16]. To give suitable care to all patients, there should be a better understanding of how antidepressants can influence glycemic control in various populations [17].

While antidepressants are commonly used to treat individuals with both depression and T2DM, there is limited evidence on how these medications affect glycemic control, particularly HbA1c levels, in large, nationally representative populations. Since many diabetics are now prescribed antidepressants, it is important to determine if the drugs improve or worsen how blood sugar is controlled [18, 19]. With National Health and Nutrition Examination Survey (NHANES) data, the study is set to discover how the use of antidepressants affects HbA1c rates in American adults living with both T2DM and depression [20, 21]. Looking at real data, the research hopes to guide clinical decisions, better handle ongoing health issues for this group, and develop treatments that suit them best. The objective of the study is to examine the association between antidepressant use and hemoglobin A1c levels in adults with comorbid T2DM and depression.

## Materials and methods

Study design and data source

This study utilized a cross-sectional design based on data from the NHANES, covering seven consecutive two-year cycles from 2005 to 2018 [21]. NHANES is a nationally representative survey conducted by the National Center for Health Statistics (NCHS), a division of the Centers for Disease Control and Prevention (CDC). It employs a complex, multistage probability sampling design that includes stratification and clustering to ensure representation of the non-institutionalized U.S. civilian population.

Data are collected through in-home interviews, standardized physical examinations conducted in mobile examination centers (MECs), and laboratory testing of biological samples. The comprehensive and standardized nature of NHANES data allows for a reliable assessment of health conditions, behaviors, and clinical outcomes across diverse population groups. The cross-sectional design of NHANES, while limiting causal inference, is well-suited for estimating national prevalence and examining associations between variables in population health research. This makes it an appropriate data source for investigating the relationship between antidepressant use, depressive symptoms, and glycemic control among U.S. adults.

Study population and inclusion criteria

This study analyzed data from adults aged 18 years and older who participated in the NHANES between 2005 and 2018 [21]. To identify individuals with probable T2DM, we employed a validated algorithm used in prior NHANES-based research. Participants were classified as having T2DM if they met all three of the following criteria: (1) a self-reported diagnosis of diabetes based on a positive response to having ever been told by a doctor or health professional that they have diabetes or "sugar diabetes"; (2) age at diagnosis of 30 years or older; and (3) did not initiate insulin therapy within one year of diagnosis, or insulin timing was missing. This operational definition helps reduce the likelihood of misclassifying individuals with type 1 diabetes. Depressive symptoms were evaluated using the Patient Health Questionnaire-9 (PHQ-9) [22], with a score of 10 or higher indicating likely depression. This threshold reflects moderate to severe symptom severity. Only those with both likely T2DM and depression were included in the analytic sample.

Variables

Outcome Variable

The primary outcome was HbA1c, expressed as a continuous percentage and measured in the NHANES laboratory component. HbA1c serves as a clinical marker of average blood glucose over the past two to three months and is a core metric in diabetes management.

Exposure Variable

This study's primary exposure of interest was antidepressant medication use, determined through self-reported prescription data collected during the NHANES medication inventory interview. Participants were classified as antidepressant users if they reported current use of one or more medications commonly prescribed for depression. Specifically, individuals were considered exposed if they reported taking any of the following agents: fluoxetine, sertraline, citalopram, escitalopram, paroxetine, venlafaxine, duloxetine, amitriptyline, or mirtazapine. These medications span multiple antidepressant classes, including SSRIs, SNRIs, and tricyclic antidepressants (TCAs). A binary variable was generated to reflect antidepressant use (1 = Yes, 0 = No), and this variable was used as the main independent variable in the analysis.

Covariates

Several sociodemographic and clinical covariates were included in the multivariable models as potential confounders, based on their established associations with both glycemic control and antidepressant use. These included age (in years), gender (male or female), and race/ethnicity (categorized as Non-Hispanic White, Non-Hispanic Black, Hispanic, or Other as referred to in the source data). Additionally, BMI, calculated as weight in kilograms divided by height in meters squared (kg/m²), was included as an important clinical factor potentially influencing HbA1c levels.

Survey weighting and handling of missing data

To ensure that estimates were generalizable to the U.S. adult population, analyses accounted for NHANES’s complex sampling design using the appropriate survey weights. Because data from seven two-year cycles (2005-2018) were combined, the two-year examination weights (WTMEC2YR) were divided by seven to generate a combined sampling weight [23]. Survey weights adjust for unequal probabilities of selection, oversampling, and survey nonresponse, ensuring that national estimates are unbiased and representative.

Variables with less than 20% missingness (age: 18.0%, HbA1c: 7.4%, BMI: 3.4%) were handled using complete-case analysis. Participants with missing data on any of these variables were excluded listwise. Although multiple imputation is widely used to address missing data, it was not employed in this study due to the complexity of the NHANES survey design. Incorporating multiple imputations while preserving design features such as survey weights, strata, and primary sampling units is statistically challenging and not directly supported by standard imputation procedures. Given the modest proportion of missingness and the adequacy of the final sample size, complete-case analysis was considered a valid and practical approach for preserving the integrity of population-level estimates.

Statistical analysis

Descriptive statistics were used to summarize the population characteristics overall and by antidepressant use status. Weighted means and standard deviations were calculated for continuous variables, while proportions were reported for categorical variables.

The association between antidepressant use and HbA1c levels was examined using multivariable survey-weighted linear regression, adjusting for all covariates listed above. Survey-weighted models were used with the svy: prefix in Stata 18 (StataCorp LLC, College Station, TX) to account for complex design. A two-tailed p-value <0.05 was considered statistically significant.

Ethical considerations

This study involved secondary analysis of publicly available, de-identified NHANES data. The National Center approved all NHANES protocols for the Health Statistics Research Ethics Review Board. Therefore, this study was considered exempt from additional institutional review board (IRB) oversight.

## Results

Descriptive statistics

Table 1 below summarizes the weighted sociodemographic and clinical characteristics of U.S. adults with comorbid T2DM and depression, stratified by antidepressant use.

**Table 1:** Weighted sociodemographic and clinical characteristics of U.S. adults with type 2 diabetes and depression, stratified by antidepressant use (NHANES 2005–2018; N = 11,141,019) Source: Compiled by the authors from NHANES survey data NHANES: National Health and Nutrition Examination Survey; N: number; Participants’ racial information is reported as documented in the source data.

Variable	No antidepressant use (N= 10399206)	Antidepressant use (N= 741813)	T-test	Chi-square	P-value
Glycated hemoglobin (HbA1c)	7.20± 1.70	7.29± 1.63	-0.68		0.5
Age (in years)	58.29± 13.17	56.25 ± 12.39	1.83		0.07
Body mass index (kg/m^2^)	37.52±9.90	37.42±8.66	0.13		0.9
Gender				2.15	0.22
Male	5050108 (93%)	400940 (7%)			
Female	5349098 (94%)	340873 (6%)			
Race/Ethnicity				11.49	0.001
Mexican American	1016075 (95%)	56828 (5%)			
Other Hispanic	771656 (96%)	32768 (4%)			
Non-Hispanic White	6538147 (92%)	555557 (8%)			
Non-Hispanic Black	1727770 (95%)	81995 (5%)			
Non-Hispanic Asian	345559 (96%)	14665(4%)			
Other Races	10399206 (93%)	741813 (7%)			

The findings from Table 1 above reveal that among the total weighted population, 11,141,019 (6.7%) reported current use of antidepressant medications. The mean HbA1c level was slightly higher in antidepressant users (M=7.29, SD=1.63) compared to non-users (M=7.20, SD=1.70), although this difference was not statistically significant (p = 0.50). Similarly, the two groups had no significant differences in BMI (kg/m²) or age (in years). Antidepressant users had a slightly lower mean age (56.25 years) than non-users (58.29 years), but this difference also did not reach statistical significance (p = 0.07). Gender distribution was similar between the groups, with no significant association observed (p = 0.22). However, a substantial difference in race/ethnicity distribution was noted (p = 0.001). Non-Hispanic White patients represented the highest proportion of antidepressant users, 555,557 (8%), compared to other racial/ethnic groups, indicating potential disparities in antidepressant use across racial backgrounds.

Multivariable linear regression

Table 2 presents findings from the survey-weighted multivariable linear regression analysis examining the association between antidepressant use and HbA1c levels, adjusting for age, gender, race/ethnicity, and BMI.

**Table 2:** Association between antidepressant use and hemoglobin A1c levels among U.S. adults with type 2 diabetes and depression: multivariable survey-weighted linear regression analysis (NHANES 2005–2018) Source: Compiled by the authors from NHANES survey data a) Standard errors in parentheses * p < 0.05, ** p < 0.01, *** p < 0.001; NHANES: National Health and Nutrition Examination Survey; Participants’ racial information is reported as documented in the source data.

Variable	Antidepressant use	Age	BMI	Gender	Race/Ethnicity	Overall
Antidepressant use (No vs. Yes)	0.091					0.118
	(0.135)					(0.138)
Age(in years)		-0.016^*^				-0.014^*^
		(0.007)				(0.006)
Body mass index (kg/m^2^)			0.015			0.013
			(0.010)			(0.009)
Gender (vs. Female)				0.171		0.037
				(0.225)		(0.224)
Race/Ethnicity (vs. Other Hispanic)					0.171	0.184
					(0.280)	(0.273)
Race/Ethnicity (vs. Non-Hispanic White)					-0.324	-0.330
-					(0.225)	(0.217)
Race/Ethnicity (vs. Non-Hispanic Black)					0.170	0.174
					(0.339)	(0.342)
Race/Ethnicity (vs. Non-Hispanic Asian)					-0.246	-0.199
					(0.378)	(0.364)
Constant	7.195^***^	8.103^***^	6.651^***^	7.114^***^	7.376^***^	7.660^***^
	(0.106)	(0.431)	(0.361)	(0.157)	(0.169)	(0.481)
Observations	2868	2868	2868	2868	2868	2868
R^2^	0.000	0.014	0.007	0.003	0.016	0.036

The results show that after adjusting for age (in years), gender, BMI, and race/ethnicity, antidepressant use was not significantly associated with HbA1c levels (beta (β) = 0.091, SE = 0.135, p > 0.05). Among the covariates, increasing age was significantly associated with lower HbA1c levels (β = -0.014, SE = 0.006, p < 0.05), suggesting better glycemic control with advancing age. BMI (kg/m²) showed a positive, though non-significant, association with HbA1c (β = 0.013, SE = 0.009). Gender and race/ethnicity did not show statistically significant associations in the fully adjusted model. The overall model explained a small proportion of the variance in HbA1c levels (R² = 0.036). These findings suggest that while demographic and clinical factors play a role in glycemic control, antidepressant use was not independently associated with differences in HbA1c among adults with T2DM and depression.

**Figure 1:**
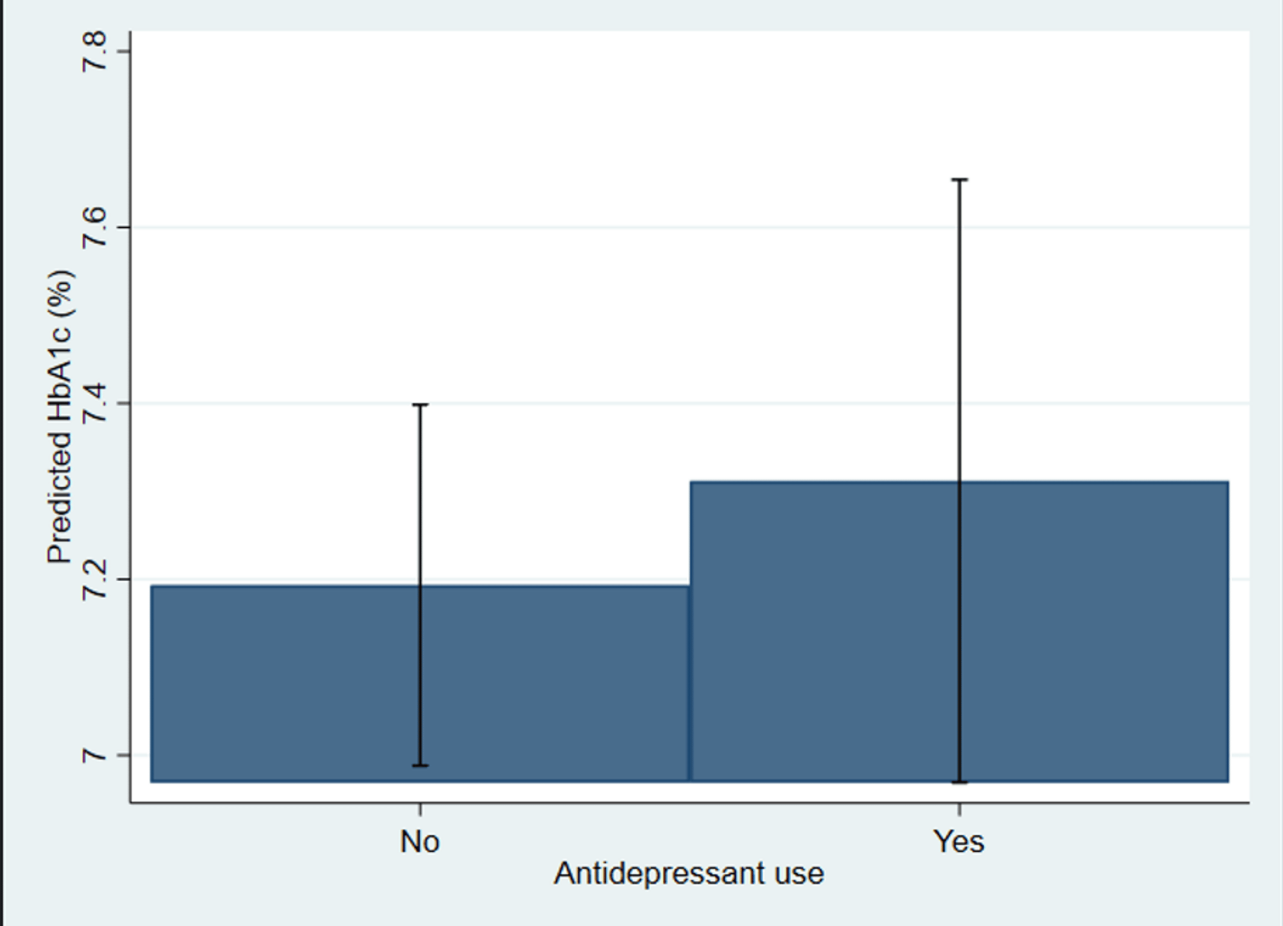
Adjusted mean hemoglobin A1c (HbA1c) levels by antidepressant use among U.S. adults with type 2 diabetes and depression (NHANES 2005–2018) NHANES: National Health and Nutrition Examination Survey

Figure 1 below illustrates the adjusted mean HbA1c levels among U.S. adults with T2DM and depression, stratified by antidepressant use, based on a survey-weighted linear regression model.

The predicted HbA1c values appear slightly higher among those using antidepressants compared to non-users. However, the difference is small and not statistically significant, aligning with the regression results. This suggests that, after adjusting for age, BMI, gender, and race/ethnicity, antidepressant use is not independently associated with elevated HbA1c levels in this population.

## Discussion

This study examined the association between antidepressant use and HbA1c levels among U.S. adults with comorbid T2DM and depression using data from the NHANES spanning 2005 to 2018. After adjusting for key demographic and clinical variables, including age, BMI, gender, and race/ethnicity, our analysis found no statistically significant association between antidepressant use and HbA1c levels. These findings suggest that antidepressant use may not have a clinically meaningful impact on glycemic control in this population.

The absence of a significant link matches results from earlier studies that also found little or no effect on blood sugar levels from antidepressant treatment in people with T2DM. Even though some antidepressants, especially tricyclics and atypical antipsychotics, are known to cause metabolic issues, the overall effect of antidepressant use in this study did not show a significant change in blood sugar levels. Such a finding may be due to the heterogeneous nature of antidepressant medications and variability in treatment duration, dosage, or adherence, none of which were captured in the NHANES dataset.

The differences seen in the backgrounds and health conditions of people who use antidepressants compared to those who don't, especially regarding race/ethnicity and gender, show how social factors influence both mental health treatment and diabetes results. For example, a larger proportion of antidepressant users were non-Hispanic White patients. At the same time, minority groups such as Mexican American and non-Hispanic Black patients were underrepresented among antidepressant users despite comparable diabetes burden. This raises concerns about potential disparities in mental health diagnosis and treatment access that warrant further exploration.

Additionally, although BMI and age were associated with HbA1c levels in the regression models, these variables did not substantially mediate the relationship between antidepressant use and glycemic control. This implies that other unmeasured factors, such as diabetes medication adherence, physical activity, diet, and psychosocial stress, could be more influential in determining glycemic outcomes.

The overall patient outcome is dependent on the level of adherence to antidepressant use. According to Hoencamp et al., antidepressant efficacy is not exclusive to the medication's pharmacological potency [24]. Several notable factors, such as patient willingness toward prescriptions and their health, medication costs, and lack of follow-up on medication use, can negatively impact their treatment goals. Not taking medication as prescribed can lead to recurring episodes of depression, and symptoms may worsen if a patient neglects personal hygiene, struggles with diet control, and has issues with glucose monitoring, which can show up as problems with glucose tolerance.

Also, it's important to note that taking antidepressants along with other medications like corticosteroids, antipsychotics, and insulin-sensitizing agents can affect blood sugar control and make it harder to understand HbA1c results in patients with T2DM. The use of corticosteroids, even at a low dose, was associated with increased HbA1c levels and increased risk of developing pre-diabetes [25]. Second-generation antipsychotics, especially olanzapine, and how long a person has been on them were the main changeable factors that could lead to T2DM in young people taking these medications [26]. This complex pharmacologic interplay can lead to fluctuating glucose levels, thereby reducing the reliability of HbA1c as a sole marker for long-term glycemic control. HbA1c levels should be monitored cautiously, with additional glycemic markers used in patients with T2DM receiving these medications.

Weight gain from antidepressants, especially from drugs like mirtazapine, tricyclics, and some SSRIs, can worsen insulin resistance and cause more fluctuations in HbA1c levels over time. Weight gain increases adiposity and disrupts glucose metabolism through alterations in the hypothalamic-pituitary-adrenal axis, adipokine release, and insulin signaling pathways [27]. In addition, these metabolic changes amplify blood glucose variations in obese or pre-obese people, compromising the stability of HbA1c, a measure that represents average glycemia over roughly three months. Long-term antidepressant treatment has been linked to elevated HbA1c and an even higher risk of developing diabetes, especially if weight gain persists. Therefore, in high-risk patients, physiologic alterations brought on by weight gain linked to antidepressants can impair long-term glycemic control and make managing diabetes risk more difficult. Furthermore, major depressive disorder (MDD) is a stress-related mental health condition that often co-exists with diabetes, affecting about 10%-15% of individuals [1, 2]. Depression worsens glycemic control in diabetics [3], and stress is linked to diabetes in both men [4, 5] and women [6, 7]. The hypothalamic-pituitary-adrenal (HPA) axis is a key regulator of the stress response, but its overactivation is implicated in MDD [8, 10], metabolic dysfunction, insulin resistance, and diabetes [9]. At the top of the HPA axis, stress triggers corticotropin-releasing hormone (CRH) release from the hypothalamus, leading to ACTH secretion from the pituitary and subsequent cortisol release from the adrenal cortex [10]. Cortisol then inhibits further HPA activation via negative feedback [10]. Cortisol mobilizes glucose, amino acids, and fatty acids for energy during stress. In skeletal muscle, it reduces insulin sensitivity, causing hyperglycemia, while in fat tissue, it increases insulin sensitivity, promoting free fatty acids and insulin resistance [9]. FKBP51, a key stress regulator, is linked to MDD [11], metabolic dysfunction, insulin resistance, and obesity [12, 13]. Elevated FKBP5 mRNA is associated with HPA axis dysfunction and poor antidepressant response in MDD [14]. Duloxetine, an SNRI, lowers FKBP5 mRNA in the ventral hippocampus and normalizes FKBP5 mRNA, protein, and GR levels in the prefrontal cortex [15]. TCAs also boost GR binding and mRNA in the hypothalamus and hippocampus, which suggests they might improve the body's response to stress hormones and help regulate the HPA axis. These findings suggest that certain antidepressants may help manage glucose dysregulation linked to HPA axis dysfunction.

Study limitations

Despite the strengths of using a large, nationally representative dataset with survey weighting to improve population-level inference, the study has limitations. Most notably, key sociodemographic variables such as education level and household income were excluded due to high levels of missing data. These factors could have provided valuable insights into socioeconomic disparities in depression treatment and diabetes management. Furthermore, the cross-sectional design of NHANES limits our ability to infer causality between antidepressant use and glycemic control. Longitudinal studies that account for the duration and type of antidepressant therapy would provide more robust evidence of the temporal effects of these medications on diabetes outcomes. Also, due to the limited nature of available data and the retrospective nature of the data used in this study, we did not factor in the mental function of the patients involved and other potential confounders such as duration of antidepressant use, medication adherence, and comorbid conditions such as hypertension, anxiety, and many others. Additionally, since variables such as physical activity and psychiatric symptom severity were not accounted for, we cannot rule out the possibility that observed associations between antidepressant use and glycemic control are influenced by behavioral or lifestyle factors rather than a direct pharmacological effect.

## Conclusions

In this nationally representative sample of U.S. adults with T2DM and depression, antidepressant use was not significantly associated with HbA1c levels after adjusting for demographic and clinical factors. These findings suggest that antidepressant use does not have a measurable impact on glycemic control in this population. Future studies should explore the role of specific antidepressant classes and consider additional social and behavioral factors that may influence diabetes outcomes.
